# World AIDS Day 2023: time to prioritize perilous HIV medicine

**DOI:** 10.1186/s13031-024-00573-w

**Published:** 2024-02-26

**Authors:** Hailay Abrha Gesesew

**Affiliations:** 1 Research Centre for Public Health, Equity and Human Flourishing, Torrens University Australia, Adelaide, Australia; 2 Tigray Health Research Institute (THRI), Mekelle, Tigray Ethiopia

**Keywords:** World AIDS Day, HIV, Conflict perilous medicine, Tigray

## Abstract

**Background:**

World AIDS Day has been observed on the first of December every year. Whilst there are specific themes during the commemoration, the role of conflict on HIV seems neglected and needs prioritization given the rise of conflicts globally.

**Discussion:**

The global HIV response brought substantial reduction of new HIV infections and HIV-related deaths, and increment of antiretroviral therapy coverage. Nevertheless, there is substantial inequity on the benefit of the response. Individuals with HIV in conflict zones have suffered immensely and are often neglected. The fact that the level, intensity, and number of conflicts is increasing mean more HIV people in conflict or post-conflict settings such as in Ethiopia, South Sudan, the Democratic Republic of Congo, Myanmar, Yemen Russia and Ukraine are at risk of negative HIV care and treatment outcomes. In particular, some conflicts such as the case of Ethiopia’s Tigray have been marked by severe public and humanitarian crises, including medical siege, intentional damage of healthcare infrastructure, targeted attacks on health workers, displacement, and appalling incidents of conflict-related sexual violence. Yet, people living with HIV in these conflict settings seem often overlooked. It is crucial to address the unique challenges in these areas to achieve the goals of AIDS/HIV care.

**Conclusion:**

There is no ideal forum to remind the intricate relationship between conflict and the HIV epidemic other than the World AIDS Day. Thus, this this year’s World AIDS Day should focus on prioritizing on tackling the direct and indirect effects of conflict on HIV transmission and treatment. This way, we can achieve the ambitious UNAIDS 95-95-95 goals and Ending AIDS by 2030.

Since 1988, World AIDS Day has been observed annually on December 1st. This day serves as a solemn reminder of the lives lost to AIDS-related illnesses, as well as a time to reflect on the progress made and to unite in enhancing our response to HIV/AIDS. Each year, World AIDS Day is centred around a specific theme. For 2023, the World Health Organization (WHO) has chosen the theme ‘Let Communities Lead.’ While the theme is broad, it underscores the importance of addressing HIV in humanitarian settings on this day. Time to prioritize perilous HIV medicine.

World AIDS Day 2023 is a time to acknowledge the mixed outcomes of the global HIV response. On a positive note, the estimated number of new HIV infections reduced by 54%, ART coverage increased from one million to 28.7 million, and HIV-related deaths of 16.5 million people were averted between 2001 and 2021 globally [1]. However, the day also brings to light the more sombre reality: individuals with HIV in conflict zones have suffered immensely and are often neglected. The year 2023 witnessed significant turmoil, including the escalation of tensions in Israel-Palestine, the ongoing Russia-Ukraine conflict, and the aftermath of the brutal 2020–2022 war in Northern Ethiopia’s Tigray region. Moreover, ongoing conflicts in South Sudan, the Democratic Republic of Congo, Myanmar, and Yemen continue to exacerbate the situation. Numerous other regions are also facing volatile conditions that could lead to further conflicts, underscoring the urgent need for attention to HIV in these crisis settings.

Some ongoing and past conflicts have been marked by severe public and humanitarian crises, including the siege of medical facilities, intentional destruction of healthcare infrastructure, targeted attacks on health personnel, widespread displacement, and appalling incidents of conflict-related sexual violence, often accompanied by the deliberate transmission of HIV [2]. A poignant example is the Tigray conflict [2], where an estimated two million people were displaced, approximately 70–80% of health facilities were deliberately damaged, health workers were targeted, and industrial-scale sexual violence against women and girls was reported. In such humanitarian crises, exacerbated by poverty, access to HIV testing, treatment, and prevention is critically hindered, leading to increased disease transmission and spread [3, 1]. Additionally, the trauma and social upheaval caused by these conflicts can lead to higher rates of risky behaviours like unprotected sex and drug use, further heightening the risk of HIV transmission.

Yet, the needs of people living with HIV, as well as HIV care continuum programs, are often overlooked in conflict settings. Addressing the unique challenges in these areas is crucial for the effective management of the global HIV epidemic. It’s imperative to establish concrete, collaborative efforts that integrate HIV prevention, testing, and treatment services into humanitarian responses in conflict zones. Additionally, addressing conflict-related sexual violence and the breakdown of social structures is essential to halt the further transmission and spread of HIV.

World AIDS Day 2023 should be a crucial reminder of the intricate relationship between conflict and the HIV epidemic. To tackle both the direct and indirect effects of conflict on HIV transmission and treatment, a concerted effort from the international community is essential. This effort should focus on designing effective, multidimensional response strategies. If perilous HIV medicine is given priority, we can achieve considerable progress in reducing the HIV burden in the world’s most vulnerable regions. Such efforts are key to fulfilling the promise of ending AIDS by 2030.

## Data Availability

All data relevant to the study are included in the article.
